# Cost of Delivering Tetanus Toxoid and Tetanus-Diphtheria Vaccination in Vietnam and the Budget Impact of Proposed Changes to the Schedule

**DOI:** 10.9745/GHSP-D-21-00482

**Published:** 2022-04-28

**Authors:** Vu Quynh Mai, Laura Boonstoppel, Kelsey Vaughan, Carl Schutte, Annette Ozaltin, Duong Thi Hong, Nguyen Mai Khanh, Hoang Manh Thang, Tran Tuan Anh, Hoang Van Minh

**Affiliations:** a Center for Population Health Sciences, Hanoi University of Public Health, Hanoi, Vietnam.; b ThinkWell, Washington, DC, USA.; c Genesis Analytics, Johannesburg, South Africa.; d National Institute of Hygiene and Epidemiology, Hanoi, Vietnam.; e Hanoi University of Public Health, Hanoi, Vietnam.

## Abstract

**Introduction:**

In 2017, aligned with global World Health Organization tetanus guidelines, Vietnam prepared evidence to support a recommendation to introduce the tetanus-diphtheria (Td) vaccine into routine immunization. This study aimed to provide evidence on the costs and budgetary impact of the potential replacement of the tetanus-toxoid (TT) vaccine with the Td vaccine, considering different possible delivery strategies.

**Method:**

We used an activity-based ingredients costing approach to estimate the 2017 program costs of providing TT vaccination to girls aged 15–16 years and conducting Td campaigns in outbreak areas. We performed a budget impact analysis for 2018–2025 using the cost per dose estimates based on the current delivery of these vaccines. We assumed complete cessation of TT vaccination of girls aged 15–16 years and a transition period where Td outbreak control campaigns would still occur. Td vaccine was assumed to be provided to children aged 7 years using either facility- or school-based delivery or combined facility- and school-based delivery.

**Results:**

The delivery cost per dose for current TT vaccination for girls aged 15–16 years was US$1.49 for school-based delivery, US$1.76 for facility-based delivery, and US$3.86 for delivery via outreach. Td vaccination through campaigns was estimated to cost US$3.56/dose. During 2018–2025, replacing the TT vaccine for girls aged 15–16 years with the Td vaccine for children aged 7 years is estimated to save US$4.61 million in immunization delivery costs if a school-based delivery strategy is used or US$1.04 million if facility-based delivery is used.

**Conclusion:**

Compared to the current plan, delivery of Td routine vaccination via a school-based strategy was the most cost saving. These results were used in late 2019 to support the delivery of Td vaccination using a school-based delivery strategy for children aged 7 years in 30 Northern provinces in Vietnam.

## Abstract (precis)

This study shows that replacing tetanus toxoid vaccination in Vietnam for girls aged 15–16 years in high-risk areas with routine tetanus-diphtheria vaccination for children aged 7 years mainly through a school-based delivery strategy will likely result in immunization cost savings.

## Teaser

**Key Findings:**

- For routine vaccination in Vietnam, we estimated that using an outreach strategy was the costliest delivery strategy to deliver the tetanus-diphtheria (Td) vaccination to children aged 7 years and tetanus toxoid (TT) vaccine to girls aged 15–16 years in high-risk areas compared to facility-based and school-based delivery of the TT vaccine.
- We estimated the budgetary impact of ceasing delivery of TT vaccines to girls aged 15–16 years in high-risk areas, introducing a dose of Td to all children aged 7 years in the country through 3 different delivery strategy options, and gradually decreasing Td campaigns in outbreak areas. We projected that if a facility-based or school-based strategy is chosen for the additional Td booster dose for all children aged 7 years, the new schedule would be less costly to the Expanded Program on Immunization than the 2017 vaccination schedule.

**Key Implication:**

- These projected cost savings must be considered with extreme caution and evaluated against the impact on coverage and equity that these changes would have. EPI must closely track surveillance data to ensure the adequacy of its proposed transition phase schedule.

## INTRODUCTION

Since the establishment of Vietnam's Expanded Program on Immunization (EPI) in 1981 and the introduction of the diphtheria-tetanus-pertussis (DTP) vaccine, the incidence of diphtheria and neonatal tetanus have reduced dramatically.^1^^,^^2^ In the 1990s, Vietnam expanded its routine immunization program for pregnant women nationwide with 3 doses of the tetanus toxoid (TT) vaccine: giving the first dose as soon as possible in pregnancy plus the second and third doses at least 1 month and 6 months after the first and the second dose, respectively, as well as giving 1 dose at the earliest contact with nonpregnant girls aged 15–16 years in high-risk areas.

Since 2005, Vietnam has declared maternal and neonatal tetanus elimination.^3^^,^^4^ A survey conducted by UNICEF, the World Health Organization (WHO), and the Government of Vietnam in 3 of Vietnam's most disadvantaged districts showed less than 1 neonatal tetanus birth per 1,000 live births. As a result, tetanus was considered eliminated in better-performing areas and was declared eliminated as a public health problem.^5^

Since then, the EPI has maintained high immunization coverage, with DTP third-dose coverage estimated at 89.0% in 2019^6^ and TT second-dose coverage for pregnant women over 87.6% in 2018.^7^ To improve lifetime protection against diphtheria and tetanus, in 2011, Vietnam added the fourth booster dose of DTP to its routine schedule to be given at 18 months.

However, over the last decade, diphtheria outbreaks have occurred more frequently in the highland and central coast regions.^8^ Cases have increased from 12 in 2012 to 21 cases in 2017.^9^ In response, EPI has immediately delivered urgent response campaigns, which provide tetanus-diphtheria (Td) vaccines for people aged 5–40 years in the outbreak areas.

In 2017, the WHO updated its recommendations for vaccination against diphtheria and tetanus. In addition to the 3 doses of DTP given to infants during their first 6 months, 3 additional booster doses of the Td-containing vaccine should be given at age 12–23 months, 4–7 years, and 9–15 years.^10^^,^^11^ In addition, WHO and UNICEF have strongly urged countries to replace the TT vaccine with the Td vaccine by 2020 to help reduce diphtheria-related morbidity and mortality.^12^ The switch to the Td vaccine is considered to have negligible financial implications because Td vaccine production is already well established internationally and domestically, and therefore does not require a huge initial investment.^12^

In 2017, in light of these recommendations, EPI was considering 2 programmatic changes. First, due to the elimination of maternal and neonatal tetanus, EPI considered recommending that Vietnam cease delivery of TT vaccines to girls aged 15–16 years in high-risk areas (Table 1). Second, EPI considered the introduction of additional Td booster doses before adulthood for all children aged 7 years to respond to occurrences of diphtheria outbreaks. These changes would also align with the global recommendation that when immediate neonatal tetanus risks are waning to shift the focus to long-term prevention of all-age tetanus across genders. Due to limited resources, Vietnam planned to start with a pilot of providing the first additional Td booster dose during 2020–2025 and eventually expand the Td vaccination schedule to include all 3 recommended boosters of Td vaccines, including the sixth dose for adolescents.

**TABLE 1.** Comparison Between WHO Recommended Tetanus and Diphtheria Vaccinations and EPI

WHO Recommendations for Routine Immunization Against Tetanus and Diphtheria	Pre-2020 EPI Immunization Schedule	Transition Period 2018–2025	Planned EPI Schedule Post-2025
DTP3: 3 doses before the age of 6	Yes	Yes	Yes
DTP4 booster: at age 12–23 months	Yes	Yes	Yes
TT during pregnancy: 3 doses for all pregnant women	Yes	Yes	Yes
TT vaccine for girls aged 15–16 years	1 dose of TT vaccine for girls aged 15–16 years in high-risk areas on a semiannual basis	Discontinued	Discontinued
Td (or diphtheria-tetanus containing) vaccine booster dose	Td campaigns targeting people aged 5–40 years in areas with diphtheria outbreaks (Td response campaigns)	A routine Td booster dose for children aged 7 years Scaling down of the Td response campaigns	A routine Td booster dose for children aged 7 years Scaling down of the Td response campaigns Planning for a Td booster dose for children aged 9–15 years (not yet decided)

Abbreviations: DTP3, third dose of diphtheria-tetanus-pertussis vaccine; DTP4, fourth dose of diphtheria-tetanus-pertussis vaccine; EPI, Vietnam Expanded Program on Immunization; Td, tetanus-diphtheria; TT, tetanus toxoid; WHO, World Health Organization.

In light of WHO and UNICEF recommendations, the Expanded Program on Immunization considered ceasing the delivery of the TT vaccine and introducing the Td vaccine.

The budgetary impact of potential changes to the immunization program is a key concern. In 2016, the total budget for the EPI was estimated at approximately US$29 million, of which 35% was funded by donors, such as Gavi, the Vaccine Alliance and UNICEF.^3^ Vietnam is expected to transition from Gavi support to fully self-finance its vaccines, hence, EPI is now demanding evidence on budget impacts. In addition, the Ministry of Health has estimated an additional US$1.08 billion resource requirement (63% from the state budget; 37% from other sources) in the coming years for vaccination against the coronavirus disease (COVID-19) for all 75 million Vietnamese. Hence, the budget for non-COVID-19 vaccines will be rigorously evaluated and will require wise planning and spending.

Therefore, to better understand the cost and budgetary impact of introducing a Td booster dose for children aged 7 years while ceasing TT vaccination of girls aged 15–16 years in selected areas, we conducted a study to estimate the delivery of TT and Td vaccination activities in 2017 and developed projects to estimate the budget impact of the proposed programmatic changes over the period 2018–2025.

### Vaccination Delivery in Vietnam

In Vietnam, routine vaccination is delivered through facility-based or non-facility-based methods, which include outreach and school-based delivery. Routine vaccination is mainly provided through facility-based delivery at commune health centers (CHCs). Because the Ministry of Health does not recommend non-facility-based vaccination, only 0.6% of communal sites in Vietnam reported doing outreach or school-based vaccination in 2017.^9^

Outreach (a non-facility-based delivery strategy) is used only in the hard-to-reach or remote areas (e.g., mountainous areas, islands). Outreach is carried out by CHC staff who travel with the vaccines, related supplies, and equipment to conduct vaccination sessions at community locations (e.g., village gathering house, village yard).

In case of using a school-based delivery strategy (non-facility-based delivery strategy), the CHC staff bring vaccines, vaccination supplies, and other medical supplies to the schools. The CHC staff are responsible for organizing the immunization sessions, including sending invitations, with support from school health staff(s) and teachers. The school medical room, which is equipped with basic medical equipment (e.g., bed, blood pressure monitor, thermometer, basic drugs), is available for such school-based vaccination events.

Previously, TT vaccination was provided to nonpregnant girls aged 15–16 years in high-risk areas with detected neonatal tetanus cases, areas with low TT vaccination coverage for pregnant women, and hard-to-reach areas (often populated by ethnic minorities) with low facility-based delivery coverage. Due to the limited resources available, TT vaccines were given only to nonpregnant girls aged 15–16 years, which, until recently, was conducted at semiannual events. The first event was often implemented via a school-based delivery strategy due to the high level of school attendance among girls aged 15–16 years, recently at 90.2%.^13^ During the second event, multiple delivery strategies might have been deployed to ensure coverage of TT vaccines for those who did not attend school. At the same time, Td rapid-response campaigns were implemented to respond to the emergency of a diphtheria outbreak. To reach the maximum coverage of Td vaccinations, multiple strategies (facility-based, school-based, and/or outreach) were often used.

## METHODOLOGY

### Study Design

We conducted a retrospective cross-sectional study to estimate the fiscal delivery costs of providing TT and Td vaccination through different delivery methods and targeting different population groups in Vietnam in 2017. We used an ingredients-based approach and classified cost activities and resource types following common methods.^14^ We took a government-as-provider perspective, including involved government immunization units from national to district level; CHCs, where the vaccination was directly delivered to the beneficiaries; and related schools, which hosted school-based vaccination events. Delivery costs per dose using different delivery strategies were then used as inputs to project the potential budgetary impact of the programmatic changes over the period 2018–2025.

### Study Sample

We used a 3-stage sampling strategy that combined purposive and random sampling, designed to be nationally representative. The sampling frame was obtained from the Ministry of Health's administrative system, which included national, provincial, district, and commune levels, as well as EPI's own regional administrative offices. First, we purposively selected the national and 3 regional-level EPI offices. At the provincial level, we purposively selected 3 of the country's 5 megacities for their geographical distribution (Hanoi in the North, Da Nang in the Center, and Ho Chi Minh City in the South). In addition, we selected 1 province in each of the 6 geographic regions based on the following criteria: (1) gross domestic product per capita near the regional average; (2) province vaccination coverage near the regional average in 2016; and (3) good health information/reporting system (based on EPI internal evaluation).

Second, we divided districts into urban and rural strata and used random sampling to select 1 urban and 2 rural districts in each province. Finally, within each selected urban district, we randomly selected 1 urban commune. Within each selected rural district, we randomly selected 2 rural communes after excluding all the township/suburban facilities. The final study frame included 73 sites: EPI national office; 3 EPI regional offices (including the south, central, and highland); 9 provincial Centers for Disease Control and Prevention (3 megacities and 6 provinces); District Preventive Medicine Center at 10 urban districts; 13 rural districts; 11 urban CHCs, and 26 rural CHCs. Among the visited sites, 18 CHCs reported having school-based vaccination and 4 CHC reported having a Td campaign for outbreak control in 2017.

### Data Collection

Data collection instruments were designed by researchers from the Hanoi University of Public Health. The Microsoft Excel-based, structured open-ended questionnaire aimed to capture the time spent on EPI activities implemented in 2017 and all related costs. The instrument was tested in 1 district in Hanoi over 2 days and revised significantly after the pilot. Data collection was carried out by 4 researchers from Hanoi University of Public Health and 3 researchers from the EPI national office who visited the sampled sites during the period May to July 2018.

The questionnaire was administered via face-to-face interviews with staff at the EPI units (from the national to district level), CHCs, and related schools. The participants were asked to estimate the number of immunization services delivered, number of working hours spent, and their additional allowance (beyond salary) received for performing each EPI activity in 2017. The activities presented in the questionnaire can be grouped as follows: (1) routine facility-based vaccination (for all available vaccines); (2) outreach vaccination (for all available vaccines); (3) semiannual school-based TT vaccination for girls aged 15–16 years if any; (4) Td response campaigns if any; (5) storage; (6) transport (collection and distribution); and (7) other activities, such as immunization safety, waste management, supervision, training, recordkeeping, cold chain maintenance, program management, and social mobilization and advocacy.

Additionally, we collected financial reports to capture shared costs (i.e., personnel cost, operational cost, maintenance cost, building and equipment costs) and the annual immunization reports of each visited site. These were standard immunization reports submitted hierarchically by all EPI units from the commune to the national level. These reports contained the number of administered doses by vaccines, fully immunized children, and TT vaccinated pregnant women and girls aged 15–16 years.

### Ethics Approval

The study design was considered and approved by the Ethical Review Board for Biomedical Research of the Hanoi University of Public Health (No. 380/2017/YTCC-HD3). Participants gave their written consent before they were interviewed. The consent documents are stored at the Hanoi University of Public Health.

### Calculating Shared Cost Per Dose

Data were entered into a Microsoft Excel spreadsheet and analyzed in Stata version 14. After data entry, we identified any abnormalities and clarified them with local staff. We allocated the joint cost to EPI activities based on annual time spent on each activity over the total amount of working time in 2017.

Some EPI activities could not be directly attributed to vaccination sessions, including storage and transport. The costs of such activities were allocated equally to all delivered doses of any vaccine and all delivery strategies. Hence, we calculated the volume-weighted averages to estimate the shared cost per dose at all levels (from national to commune).

#### Cost Per TT Dose to Girls Aged 15–16 Years Via Facility-Based or Outreach Strategy

Unfortunately, the CHC recorded only aggregated monthly vaccine delivery data that were not disaggregated by delivery strategy (facility-based or outreach). Hence, we estimated the delivery cost per dose of all routine vaccines across delivery strategies and could not calculate the delivery cost for facility-based and outreach delivery separately. The assumption in the share of delivered doses between facility-based and outreach delivery strategies was taken from interviews with CHC staff, which showed the share at 69% and 31%, respectively. This assumption then was used to calculate doses of TT to girls aged 15–16 years delivered via facility-based or outreach strategy. Hence, the final cost per dose of TT to girls aged 15–16 years via facility-based strategy was calculated as the sum of the shared cost per dose and a share of the total cost of the group of activities related to routine facility-based vaccination, and total estimated doses delivered via facility-based strategy. We applied the same method to estimate the cost per dose of TT to girls aged 15–16 years via outreach strategy.

#### Cost Per TT Dose to Girls Aged 15–16 Years Via School-Based Strategy

The events of school-based TT vaccination for girls aged 15–16 years were often performed separately from the routine vaccination. Thus, the cost per dose of TT to girls aged 15–16 years via a school-based strategy was calculated directly using total costs for the group semiannual school-based TT vaccination for girls aged 15–16 years and total TT dosed delivered in such school-based events. The shared cost per dose was then added on to make the final cost per dose of TT to girls aged 15–16 years using a school-based strategy.

#### Cost Per Dose of Delivering Td Response Campaigns

The Td rapid-response campaigns were conducted independently, hence, separate data for Td response campaigns could be obtained. The cost per Td campaign dose was calculated using total costs at group “Td response campaigns” and total Td dosed delivered in the outbreak-controlled campaigns plus the shared cost per dose.

### Projecting the Potential Programmatic Changes

In addition to estimating the costs of the existing schedule, we estimated the budgetary impact of the cessation of the TT vaccination for girls aged 15–16-years in high-risk areas and the introduction of an additional booster dose of Td vaccine for boys and girls aged 7 years. Following discussions with EPI, we assumed that these programmatic changes would be introduced over a transition period covering 2018–2025, whereby potential diphtheria-control campaigns would be continued until high coverage of Td vaccination of children aged 7 years is reached. We assumed that following an evaluation of this transition phase, Vietnam would add the additional recommended Td booster dose for all adolescents to its schedule as well, depending on the availability of resources (Table 1).

When estimating the budgetary impact of the vaccine schedule change, we assumed that these changes would be introduced from 2018–2025, whereby potential diphtheria-control campaigns would be continued until high coverage of Td vaccination is reached.

The cost of the new schedule was estimated using cost assumptions based on the cost estimates of the schedule that was in place in 2017. Three possible delivery scenarios were identified by EPI for the new schedule of Td vaccine: (1) using facility-based delivery only, (2) a mixed strategy using both facility-based delivery and outreach, and (3) school-based delivery only. For the second scenario, the proportion of doses to be delivered via facilities as compared to outreach was based on 2017 delivery volumes via these strategies.

The budgetary impact was estimated as the difference between a scenario in which the current schedule would be maintained, and the cost of a transition period during which TT vaccination for girls aged 15–16 years in high-risk areas would cease, a booster dose of Td vaccine would be introduced for all children aged 7 years, and Td campaigns in response to diphtheria outbreaks would be gradually less and less required. We only estimated the cost of the 2 schedules and not a potential change in cost-effectiveness. We did not evaluate the impact that the change in the vaccine schedule, target population, and delivery strategy coverage could have on coverage and equity.

### Budgetary Impact Analysis of Potential Programmatic Changes

For the cost projection of maintaining the existing 2017 schedule, we estimated that the number of girls aged 15–16 years in high-risk areas was 1,286,634 in 2018^13^ and assumed to increase by 1% population growth each year. The number of Td doses needed for outbreak control campaigns was based on the reported number of doses used in 2017 (82,603 doses) and corrected for 1% population growth, as estimated by the General Statistics Office.^13^ For the cost projection of the proposed introduction of a Td booster dose for children, we assumed that the target population was equal to the number of children aged 7 years in 2018 (1,697,105 children) and assumed to increase by 1% each year.^13^

We assumed that the coverage under the existing schedule would remain constant at 2017 levels. For the new schedule, we assumed a gradual uptake of the Td booster dose, given the constraint that EPI would only be able to provide 800,000 Td doses in 2018. This meant that only 50% of all children aged 7 years could receive this booster dose in 2018. Following that, we assumed that the uptake of the new Td booster dose would increase by 10% annually, reaching full uptake at current coverage levels (90%) by 2022. The rollout of the Td booster dose would be prioritized in provinces that were most prone to diphtheria outbreaks, so we assumed that the probability of having a diphtheria outbreak would be reduced by 50% compared to 2017 levels. By 2022, once the Td booster would be rolled out nationwide, we assumed Td outbreak response campaigns would no longer be required. Table 2 shows the target populations and uptake and coverage estimate for the budget analysis.

**TABLE 2.** Projected Targeted Population and Coverage Assumption for Tetanus Toxoid Vaccine and Tetanus-Diphtheria Vaccine Campaign in Vietnam

	Maintaining the 2017 Schedule				Changes in Vaccination Schedule			
	One Dose of TT Vaccine Girls Aged 15–16 Years in High-Risk Areas on a Semiannual Basis		Td Diphtheria Outbreak Response Campaigns Targeting People Aged 5–40 Years		One Dose of Td Vaccine for Children Aged 7 Years		Transition for Td Diphtheria Outbreak Response Campaigns^a^	
	Projected Targeted Population	Coverage Assumption	Projected Targeted Population	Coverage Assumption	Projected Targeted Population	Coverage Assumption	Projected Targeted Population	Coverage Assumption
2017 (present study)	1,100,000		82,603				82,603	
2018	1,286,634	100%	83,429	100%	1,697,105	50%	83,429	50%
2019	1,299,500	100%	84,263	100%	1,714,076	60%	84,263	50%
2020	1,312,495	100%	85,106	100%	1,731,217	70%	85,106	50%
2021	1,325,620	100%	85,957	100%	1,748,529	80%	85,957	50%
2022	1,338,876	100%	86,817	100%	1,766,014	90%	86,817	50%
2023	1,352,265	100%	87,685	100%	1,783,674	100%	87,685	0%
2024	1,365,788	100%	88,562	100%	1,801,511	100%	88,562	0%
2025	1,379,446	100%	89,448	100%	1,819,526	100%	89,448	0%

Abbreviations: Td, tetanus-diphtheria; TT, tetanus toxoid.

a This transitional period aims to help control diphtheria outbreaks while the new schedule of Td vaccine for children is still to be fully implemented.

### Sensitivity Analysis

We intended to capture the uncertainty of budget impacts for different delivery platforms of the Td vaccine schedule change. First, we estimated the budgetary impact of a 7% increase in health worker salaries, based on the Government roadmap on basic wage adjustments. Second, allowances for staff (e.g., travel, training) were not fully covered due to the limited budget, and we estimated the budgetary impact of providing full allowances for such activities. An additional scenario was added in the sensitivity analysis where the coverage of the Td vaccine for diphtheria outbreak response was gradually reduced by 10% yearly (starting at 50% in 2018 and reaching 0% in 2023).

## RESULTS

All findings are volume-weighted averages of the fiscal costs of TT and/or Td delivery and are presented in 2018 US$. Costs were adjusted to 2018 price levels using Vietnam's commercial price index.^6^

Total expenditure on immunization in 2017 of the visited sites had been recorded. The total cost of supply storage, transportation, training, recordkeeping (data management), and supervision had a similar share (about 15%–20% of the total cost) at the national, regional, and provincial levels. At the district level, the supervision activities were a majority of the costs (55.6% of the total cost). At the commune level, the main costs (about 70% of the total cost) were from vaccination (Table 3). Among cost inputs for vaccination, the cost input for personnel was more than 70% of the total cost at the district and facility levels, whereas the percentages of personnel, transport, and cold chain were more significant (15% and more of the total cost) than the others at the national, regional, and provincial levels (Table 3). The total cost was allocated per dose using the volume of delivered vaccines with each strategy.

**TABLE 3.** Percentages of the Fiscal Cost Components TT and Td Delivery and Cost Inputs by Levels of Implementation

	By Level of Implementation				
	National Level	Average Regional	Average Provincial	Average District	Average Facility
Average total cost 2018 US$	749,363	75,158	41,044	7,720	2,693
Cost Components					
Facility-based vaccination^a^	0.0%	0.0%	0.0%	0.0%	62.8%
Outreach vaccination^b^	0.0%	0.0%	0.0%	0.0%	7.9%
School-based vaccination^c^	0.0%	0.0%	0.0%	0.0%	1.2%
Td campaign^d^	0.0%	0.0%	2.5%	2.4%	3.4%
Supply storage	32.4%	10.8%	5.9%	5.6%	2.5%
Transport	20.8%	17.8%	7.3%	10.8%	6.8%
Immunization safety	3.4%	1.8%	4.1%	0.3%	0.0%
Waste management	0.0%	0.0%	0.3%	0.2%	0.7%
Supervision	13.6%	22.1%	22.6%	55.6%	0.4%
Training	10.4%	7.8%	24.6%	3.7%	1.8%
Recordkeeping	12.6%	13.6%	17.4%	11.2%	6.4%
Cold chain maintenance	2.9%	18.3%	5.0%	2.2%	1.2%
Program management	3.0%	6.9%	5.7%	6.3%	4.5%
Social mobilization and advocacy	0.8%	1.0%	4.5%	1.6%	0.9%
Cost Inputs					
Salaried labor	14.2%	26.2%	27.7%	72.9%	83.0%
Per diems	1.7%	15.9%	13.9%	7.8%	4.5%
General supplies	1.8%	0.0%	2.1%	2.3%	1.4%
Transport and fuel	14.7%	28.8%	9.4%	2.8%	0.2%
Vehicle maintenance	0.2%	2.0%	0.1%	2.4%	0.0%
Cold chain energy costs	2.9%	5.7%	4.4%	0.6%	1.2%
Printing	9.0%	2.4%	12.5%	0.1%	0.0%
Building overheads	5.1%	1.9%	2.4%	4.7%	2.6%
Other recurrent	23.1%	16.9%	23.0%	2.5%	2.5%
Vehicles	0.0%	0.0%	0.0%	0.0%	0.0%
Cold chain equipment	27.2%	0.2%	2.0%	1.5%	0.8%
Buildings	0.0%	0.0%	0.0%	0.0%	0.5%

Abbreviations: Td, tetanus-diphtheria; TT, tetanus toxoid.

a Routine facility-based vaccination for the TT vaccine for girls aged 15–16 years.

b Routine outreach vaccination for the TT vaccine for girls aged 15–16 years in some remote areas.

c Non-routine school-based vaccination events for the TT vaccine for girls aged 15–16 years.

d Td vaccination campaign for adults in outbreak areas.

### Cost of the 2017 TT/Td Immunization Schedule

The cost of delivering a TT dose to girls aged 15–16 years was greatest through outreach (US$3.86), followed by facility-based (US$1.76) and school-based delivery (US$1.49) (Table 4). The higher cost of outreach is due to higher related travel costs and shared costs (the latter allocated based on the greater amount of time spent on outreach-based vaccination). For Td campaigns in responses to diphtheria outbreaks, the delivery cost per dose was lower than routine outreach but higher than facility-based or school-based delivery of TT (US$3.56).

**TABLE 4.** Average Cost Per Vaccine Dose Delivered in Vietnam Using the 2017 Schedule

Delivery Strategy	No. of Facilities	No. Doses Delivered in 2017 (%)	Fiscal Cost of Delivery		
			Cost Per Dose, 2018 US$	Standard Error	95% CI
**Routine: TT vaccination for girls aged 15–16-years in high-risk areas**		1,100,000 (100)			
**Facility-based vaccination**	37	305,723 (27.8)	1.76	0.01	1.74, 1.79
**Outreach vaccination**	5	137,354 (12.5)	3.86	0.12	3.63, 4.09
**School-based vaccination**	18	656,923 (59.7)	1.49	0.02	1.46, 1.52
**Td vaccination through campaigns**	4	82,603 (100)	3.56	0.06	3.35, 3.57

Abbreviations: CI, confidence interval; Td, tetanus-diphtheria; TT, tetanus toxoid.

For the 2 delivery strategies that were used in both rural and urban areas, facility-based and school-based delivery, the cost per dose delivered via facility-based strategy was US$0.11 higher at rural facilities. Outreach and campaigns were only used in rural areas, with a much higher cost per dose (US$3.56–US$3.59) than any of the other delivery strategies used in either urban or rural areas (Table 5). The higher costs in rural areas were mainly due to the additional support provided by the Government of Vietnam and EPI to rural facilities in the form of higher performance-related bonuses and/or higher monthly staff salaries.

**TABLE 5.** Cost Per Vaccine Dose Delivered by Delivery Strategy, Location, and Level of the Health System in Vietnam

	Fiscal cost per dose (2018 US$)					
	Facility-Based Delivery		School-Based Delivery		Outreach	Campaign
	TT Vaccine		TT Vaccine		TT Vaccine	Td Vaccine
	Urban	Rural	Urban	Rural	Rural	Rural
No. of facilities/communes	11	26	3	15	5	4
Joint cost per dose						
National level	0.03	0.03	0.03	0.03	0.03	0.03
Regional level	0.05	0.05	0.05	0.05	0.05	0.05
Provincial level	0.07	0.06	0.08	0.07	0.07	0.07
District level	0.32	0.25	0.36	0.28	0.28	0.28
Commune level	0.28	0.37	0.28	0.37	0.44	1.39
Vaccination session cost						
Commune level	0.95	1.04	0.73	0.65	2.99	1.74
Total cost per dose	1.70	1.81	1.53	1.45	3.86	3.56

Abbreviations: Td, tetanus-diphtheria; TT, tetanus toxoid.

**FIGURE 1:**
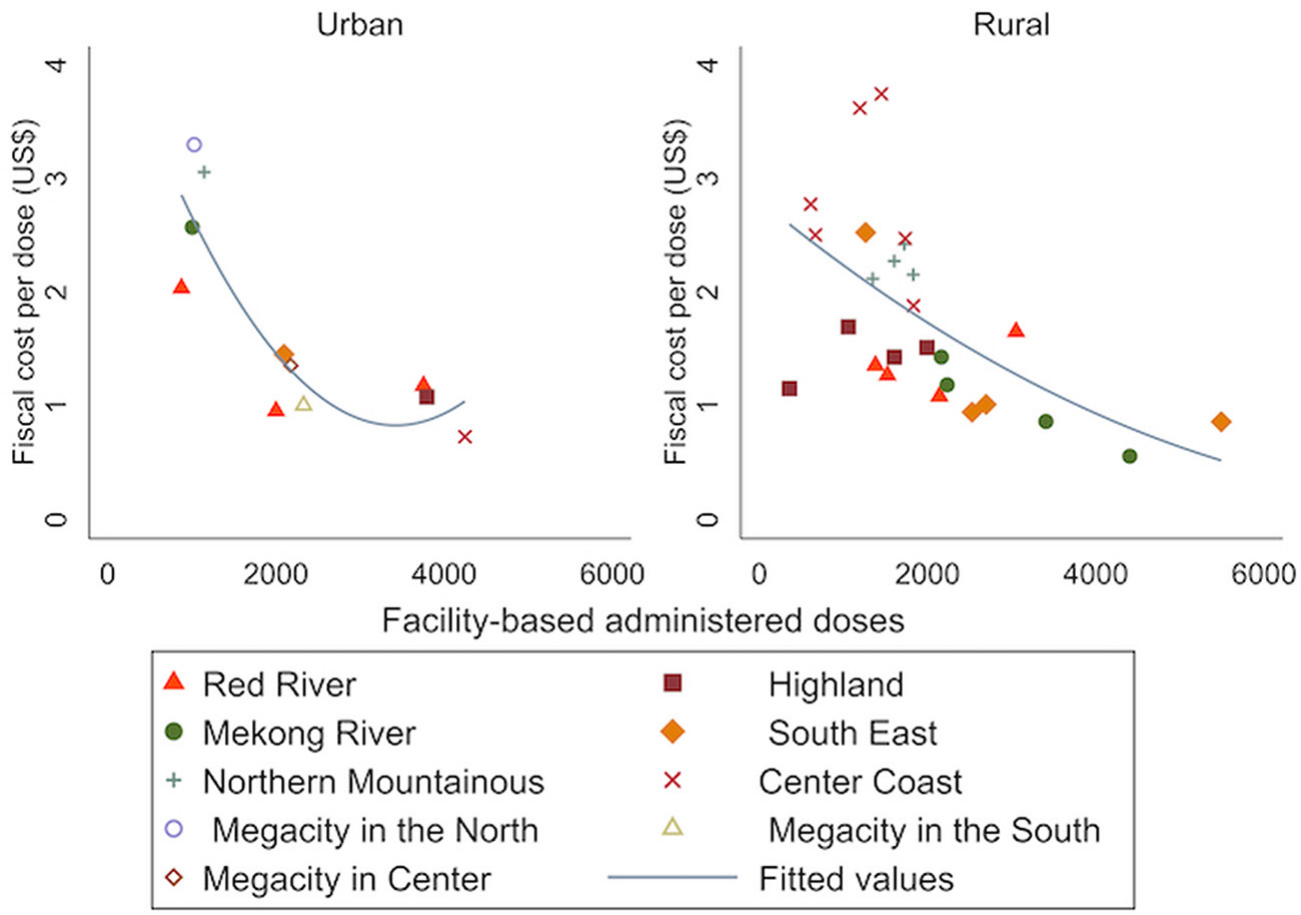
Cost Per Dose for Tetanus Toxoid Vaccination for Girls Aged 15–16 Years via Facility-Based Strategy by Region in Vietnam *Costs were adjusted to 2018 prices using Vietnam's consumer price index.

In both urban and rural areas and across all regions, the fiscal cost per dose was lower in CHCs that delivered a greater number of doses (Figure 1). This confirms the inverse relationship between the cost per dose and the volume delivered that has been proven widely in several economic immunization studies.^15^^,^^16^ Among the urban CHCs, the highest cost per dose with a facility-based strategy was seen in Hanoi, the megacity in the north (Figure 1). Among rural areas, the sampled province in the central coast had the highest cost per facility-based dose in the sample. This province is currently a priority province by the Government and EPI and therefore, has received various forms of financial support resulting in a higher cost per dose. We also noted differences in the cost per dose delivered through Td campaigns between the highlands (US$2.37) and central coast area (US$4.57). The higher-than-average cost per dose in the northern mountainous areas is also due to the increased government support for this region. The Supplement Tables present detail on percentages of the cost components and cost inputs by geographical region.

In both urban and rural areas and across all regions, the fiscal cost per vaccine dose was lower in CHCs that delivered a greater number of doses.

### Budgetary Impact of the Proposed Change in Schedule

**FIGURE 2:**
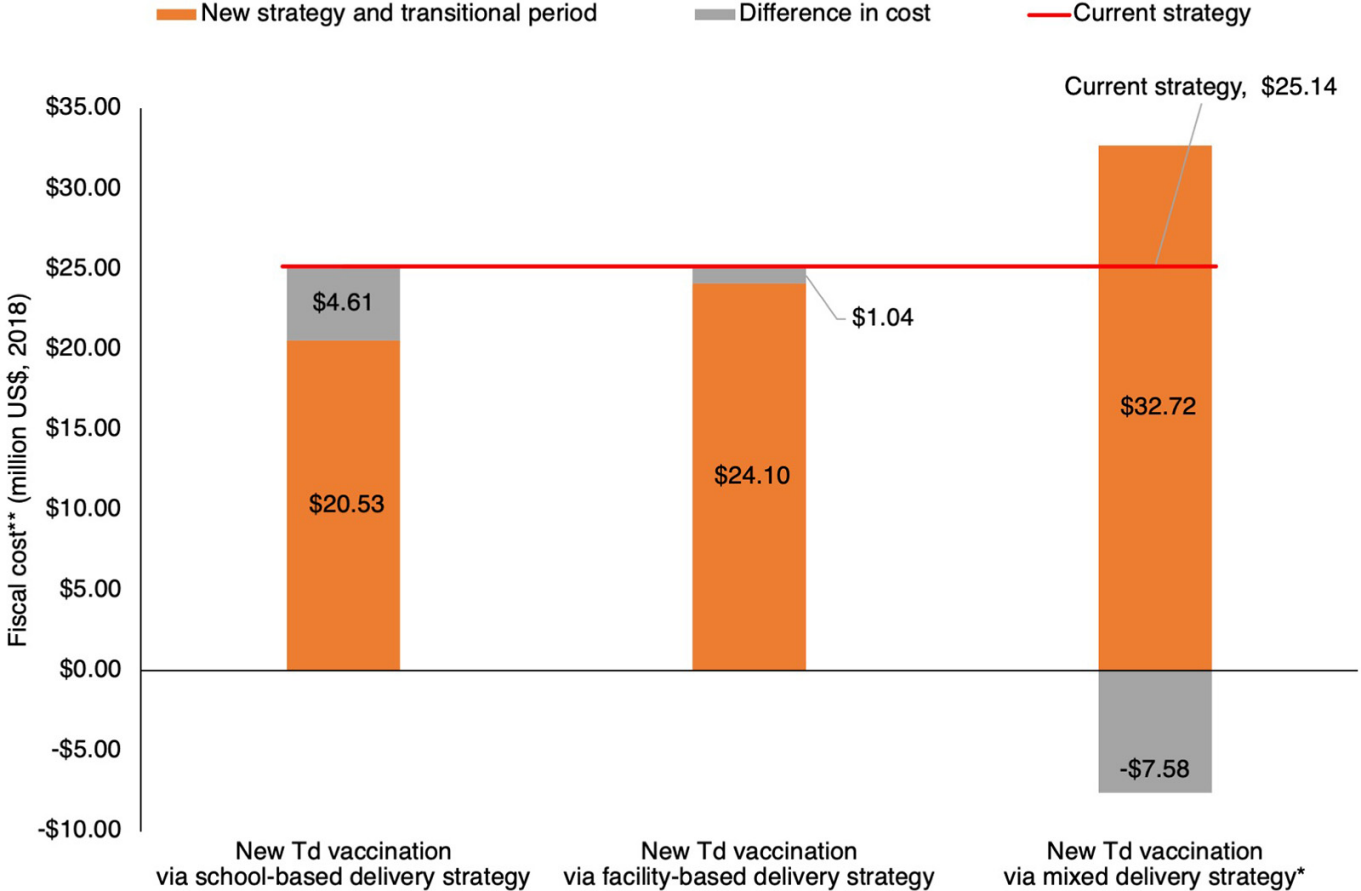
Total Cost of Replacing TT Delivery to Girls Aged 15–16 Years With Td Delivery to Children Aged 7 Years During 2018–2025, Vietnam Abbreviations: Td, tetanus-diphtheria; TT, tetanus toxoid. *The mixed delivery strategy refers to 69% facility-based and 31% outreach strategy. **Costs were adjusted to 2018 prices using Vietnam's consumer price index.

Figure 2 shows the total costs of keeping the TT/Td immunization schedule that was in place in 2017 compared to the new schedule proposed for the 2018–2025 transition period, using 3 potential delivery strategy options. Maintaining the cost of the 2017 schedule for the entire 2018–2025 period was estimated at US$25.14 million. Using facility-based delivery only, the total cost of the new schedule (US$24.10 million) would be lower than that, resulting in a cost savings of US$1.04 million. Using a mixed delivery strategy (facility-based and outreach), the total cost of the new schedule is estimated at US$32.72 million or US$7.58 million higher than maintaining the 2017 schedule. Using the school-based delivery strategy projected the greatest cost saving at US$4.61 million for the period 2018–2025 compared with keeping the current schedule. Details of the estimated total costs and cost savings from the new Td vaccination schedule by each scenario are included in Supplement Figures 1a, 1b, and 1c.

### Sensitivity Analysis

The Supplement Figures 2a, 2b, 2c present the budget impact if the cost per dose were increased due to a hypothetical 7% increase in health worker salaries (scenario 1), paying full allowances for staff (e.g., travel, training) (scenario 2), and a 10% annual reduction in Td response campaigns (scenario 3), respectively. Regardless of the changes in cost per dose or number of Td doses delivered in the response campaign, the mixed delivery platform—combining 69% of doses delivered in facilities and 31% of doses delivered in outreach sites—was always the most expensive strategy. However, the magnitudes in differences between the 2017 schedule and the proposed schedule were much smaller for the mixed delivery platform in case of higher basic salary levels and full allowances for staff.

## DISCUSSION

In this study, we estimated the cost of the TT/Td delivery schedule in place in 2017, including TT vaccine delivery to girls aged 15–16 years in high-risk areas, and Td campaigns in areas where diphtheria outbreaks have occurred. We showed that in 2017, outreach was the costliest delivery strategy (US$3.86 per dose) followed by facility-based delivery (US$1.76 per dose) and school-based delivery (US$1.49 per dose). The main reasons for this difference are the inverse relationship between the cost per dose delivered and the volume delivered at each of the sites, which is commonly seen in immunization costing literature, and the additional labor and transport costs related to outreach. Outreach is utilized only in rural remote areas with small population sizes and represented less than 4% of doses delivered in our sample. The cost of Td vaccination through campaigns was estimated at US$3.56 per dose. The outbreak responses required higher transport and fuel costs when compared to TT delivery as there is greater movement of staff required during campaigns to deliver vaccines to the rural communities that are targeted.

We noted variations in the cost per dose of routine TT and Td delivery between 6 geographical regions, even across areas where the number of doses delivered was similar. This was expected given the different levels of funding for EPI at the provincial and district levels, reflecting each local authority's priorities and their budget capacity. For example, the costs were higher in the northern mountainous areas due to higher levels of government support for salaries and bonuses for this region. In Hanoi, the city had prepared funding to fully compensate the bonus for CHC staff, which led to a much higher facility-based cost per dose in Hanoi compared to other megacities.

These differences between regions were also visible in the cost of delivery through Td campaigns. In the highlands, the outbreak had already been controlled at the time of vaccination, thus, the area received far less support from the government for the campaign and delivered fewer doses compared to the campaign in the central coast area. The cost per dose was slightly different between rural and urban sites. Due to the higher number of delivered doses via the facility-based strategy in urban areas (average of 2,223 doses/site compared to 1,972 doses/site in rural ones), urban areas reported a lower facility-based cost per dose. In contrast, the number of doses delivered via the school-based strategy was higher among rural sites (71 doses/site among rural sites compared to 45 doses/site in urban sites), leading to a lower cost per dose via the school-based strategy in rural areas. In addition, the government often suggests a higher bonus for staff working in hard-to-reach settings (e.g., mountainous, rural, remote areas), which also contributed to a higher facility-based cost per dose in rural areas compared to urban.

Limited evidence exists of the cost of changing TT/Td vaccination and the delivery cost of different strategies. However, we can compare the unit cost findings from this study to the closest comparable global evidence on immunization delivery costs for other vaccines. The results presented in our study were in line with results found from a review for delivering maternal immunization during pregnancy.^17^ In addition, we converted our study findings into 2016 US$ for comparability with the global evidence in the Immunization Delivery Cost Catalogue.^18^ For school-based delivery, the closest comparison from the global evidence is for school-based delivery of the human papillomavirus (HPV) vaccine, which is estimated at US$1.74 to US$2.24 per dose (financial costs, excluding vaccine costs). This is higher than our estimate of US$1.42 (fiscal cost, excluding vaccine costs) for school-based delivery of the Td vaccine, especially considering that the global estimate only includes incremental costs.^19^ Other reasons for the difference may be related to country contexts. Vietnam's higher population density means a reduced distance to health facilities and vaccination sites compared to the more sparsely populated countries of Lao People's Democratic Republic, Peru, and Uganda, from which the global estimates are drawn. Differing salary levels and the degree to which services are integrated are also factors that influence delivery costs.^20^ However, it should also be noted that the delivery of HPV vaccine was on a pilot/demonstration project basis where costs may be higher than at full-scale implementation; estimates of the cost of HPV vaccine delivery at full-scale implementation are likely to be lower.

There are not enough comparable cost estimates from the literature on campaign delivery to be able to make a comparison with this study's findings. Our findings are also not comparable with previous costing studies in Vietnam that examined different types of costs and vaccines, some on a demonstration basis. These studies estimated the full and incremental financial and economic costs of delivering specific vaccines (Bacillus Calmette-Guérin, DTP, measles, oral polio vaccine, TT) and a schedule of vaccines to children under age 5 years using facility-based delivery in 1 specific northern province as well as other various districts in the country.^21^^,^^22^

At EPI's request, this study has estimated the budgetary impact of a potential change in the TT/Td vaccination schedule. We projected the cost of ceasing delivery of TT vaccines to girls aged 15–16 years in high-risk areas, the introduction of a Td dose to all children aged 7 years in the country through 3 different delivery strategy options, and a gradual scale down of Td campaigns in outbreak areas. Our findings show that if a facility-based or school-based strategy is chosen for the additional Td booster dose for children, the new schedule would be less costly to EPI than the 2017 vaccination schedule. The new schedule would only be more costly in case of a mixed delivery strategy whereby 31% of the doses would be delivered through outreach and the remainder at facilities. Additionally, if Td outbreak response campaigns would continue to be needed in the future, the new schedule may also continue to be more costly.

However, these projected cost savings must be evaluated against the impact on coverage and equity that a change in the vaccination schedule would have. Assuming outbreak response campaigns with Td vaccine would no longer be required is a strong assumption, and the need for semiannual TT vaccination for girls aged 15–16 years in high-risk areas is not necessarily offset by the introduction of a Td booster dose for children aged 7 years. The new schedule proposed in this study and that was being evaluated by EPI under the assumption that fiscal growth for immunization and TT/Td vaccination specifically would be limited over the coming years hinders the possibility of adding the additional WHO-recommended Td booster dose for adolescents. This is still envisioned post-2025 if funding allows. In the meantime, EPI must closely track surveillance data to ensure the adequacy of its proposed transition phase schedule. It must ensure that an immunity gap does not develop if school-based vaccination rates are unable to make up for the discontinuing of TT vaccination for girls aged 15–16 years in high-risk areas.

These projected cost savings must be evaluated against the impact on coverage and equity that a change in the vaccination schedule would have.

### Limitations

There are several limitations of this study relating to the data used. First, some data were missing or sparse, such as the number of doses delivered at outreach vaccination sessions. We had to make some assumptions, for example, using percentage allocations from similar facilities. Second, in some cases, we found inconsistencies in recordkeeping on the number of administered doses, with different numbers recorded at the facility and district levels. We presented the inconsistencies to both CHC and district staff, who we asked to select the number they thought to be most accurate. Regardless of these limitations, given our large sample size and the representative design, as well as the fact that we validated our findings with both EPI staff and managers, we feel confident with the results presented here. Nevertheless, we have not included in our analysis the broader health care costs and other economic consequences associated with the burden from diphtheria or a potential resurgence of tetanus, thus our results do not reflect the full picture of the impact of a change in the TT/Td vaccination schedule. Evidence on the economic burden related to diphtheria has not been well documented in Vietnam. Further studies should be done to provide the epidemiological evidence that should support the costing study.

## CONCLUSION

The study results show that replacing the TT vaccine for girls aged 15–16 years in high-risk areas with routine Td vaccination for children aged 7 years mainly through a school-based delivery strategy will likely result in immunization cost savings. This recommendation is appropriate given that 97.9% of children aged 7 years attend primary school in Vietnam.^13^ According to EPI, the results have already been used for the pilot of delivery of Td to children aged 7 years during the last quarter of 2019. EPI used both facility- and school-based delivery strategies, which our study showed were the least costly delivery methods. The results should nevertheless be considered with caution and evaluated next to impact and surveillance data.

## Supplementary Material

GHSP-D-21-00482-supplement.pdf

## Notes

**Peer Reviewed**

**First published online:** April 5, 2022.
