# Supplementary material for: Cost of Delivering Tetanus Toxoid and Tetanus-Diphtheria Vaccination in Vietnam and the Budget Impact of Proposed Changes to the Schedule
Source: Glob Health Sci Pract. 2022 Apr 28;10(2):e2100482. doi: 10.9745/GHSP-D-21-00482 (PMC9053159; doi:10.9745/GHSP-D-21-00482)
Supplement: GHSP-D-21-00482-supplement.pdf [file GHSP-D-21-00482-supplement.pdf]

Supplement to: Mai VQ, Boonstoppel L, Vaughan K, et al. Cost of delivering tetanus toxoid and tetanus-diphtheria vaccination in Vietnam and the budget impact of proposed changes to the schedule. *Glob Health Sci Pract*. 2022;10(2): e2100482. https://doi.org/10.9745/GHSP-D-21-00482

Supplement Table A. Percentages of the fiscal cost components by geographical regions of implementation

	By geographical region of implementation								
	Average in South East	Average in Red river	Average in Northern mountainous	Average in MeKong River	Average in Megacity in the South	Average in Megacity in the North	Average in Megacity in Centra	Average in Highland	Average in Coastal center
Facility-based vaccination [1]	5.7%	1.2%	8.8%	13.0%	1.4%	1.6%	2.2%	8.1%	11.4%
Outreach vaccination [2]	0.0%	0.0%	5.8%	0.0%	0.1%	0.0%	0.0%	2.3%	0.0%
School-based vaccination [3]	0.2%	0.0%	0.4%	0.4%	0.0%	0.0%	0.0%	0.0%	0.2%
Td campaign [4]	0.0%	0.0%	0.0%	0.0%	0.0%	0.0%	0.0%	1.5%	11.9%
Supply storage	14.3%	30.5%	4.6%	2.5%	8.1%	3.9%	1.8%	3.3%	3.5%
Transport	14.0%	19.6%	9.1%	6.2%	18.2%	1.1%	6.9%	22.8%	11.8%
Immunization safety	2.0%	3.2%	0.0%	0.1%	1.0%	9.5%	0.0%	0.5%	0.7%
Waste management	0.0%	0.0%	0.4%	0.3%	0.0%	0.0%	0.1%	0.1%	0.9%
Supervision	18.9%	15.8%	24.3%	29.0%	18.6%	26.0%	48.6%	24.7%	29.7%
Training	13.5%	11.2%	8.7%	15.8%	9.4%	32.3%	6.7%	5.0%	1.8%
Record keeping	13.8%	11.8%	14.2%	21.0%	21.1%	16.9%	15.4%	14.8%	9.1%
Cold chain maintenance	11.4%	3.0%	5.3%	1.0%	7.8%	5.6%	1.1%	12.7%	9.7%
Program management	5.3%	3.0%	6.8%	9.9%	13.8%	2.8%	3.4%	3.1%	8.8%
Social mobilization & advocacy	0.9%	0.8%	11.5%	0.9%	0.4%	0.1%	13.9%	1.1%	0.5%

Notes: [1]: Routine facility-based vaccination of TT vaccine for girls aged 15–16 years. [2]: Routine vaccination for the TT vaccine for girls aged 15–16 years delivered via outreach in some remote areas. [3]: Non-routine school-based vaccination events for the TT vaccine for girls aged 15–16 years. [4]: Td vaccination campaign for adults in outbreak areas.

Supplement to: Mai VQ, Boonstoppel L, Vaughan K, et al. Cost of delivering tetanus toxoid and tetanus-diphtheria vaccination in Vietnam and the budget impact of proposed changes to the schedule. *Glob Health Sci Pract*. 2022;10(2): e2100482. https://doi.org/10.9745/GHSP-D-21-00482

Supplement Table B. Percentages of the fiscal cost inputs by levels and geographical regions of implementation

	Salaried labor	Per diems	General supplies	Transport & fuel	Vehicle maintenance	Cold chain energy costs	Printing	Building overheads	Other recurrent	Vehicles	Cold chain equipment	Buildings
Red river	18.6%	1.8%	1.8%	13.5%	0.2%	2.9%	8.2%	5.0%	22.3%	0.0%	25.5%	0.1%
Highland	52.1%	10.1%	0.9%	18.6%	2.9%	3.5%	6.2%	3.2%	0.6%	0.0%	0.3%	0.1%
MeKong River	56.0%	10.5%	0.8%	3.0%	0.1%	0.7%	10.4%	2.4%	15.4%	0.0%	0.7%	0.0%
South East	27.2%	16.0%	0.9%	29.0%	1.2%	0.2%	0.1%	1.8%	23.6%	0.0%	0.1%	0.0%
Northern mountainous	56.1%	7.4%	1.3%	7.2%	3.4%	2.2%	14.6%	2.0%	4.3%	0.0%	1.4%	0.0%
Coastal center	56.4%	10.6%	0.7%	5.5%	0.2%	8.2%	1.3%	3.0%	1.1%	0.0%	1.1%	0.0%
Megacity in the North	22.8%	24.7%	0.1%	8.3%	0.0%	5.6%	14.5%	2.3%	21.5%	0.0%	0.1%	0.0%
Megacity in the South	27.9%	3.7%	1.1%	2.3%	0.5%	6.1%	0.7%	5.9%	51.6%	0.0%	0.0%	0.0%
Megacity in Centra	50.7%	5.0%	12.5%	15.9%	0.3%	1.1%	6.9%	3.0%	4.4%	0.0%	0.3%	0.0%

Supplement to: Mai VQ, Boonstoppel L, Vaughan K, et al. Cost of delivering tetanus toxoid and tetanus-diphtheria vaccination in Vietnam and the budget impact of proposed changes to the schedule. *Glob Health Sci Pract*. 2022;10(2): e2100482.
https://doi.org/10.9745/GHSP-D-21-00482

Supplement Figure 1a. Estimated cost savings from the new schedule for Td vaccine to children aged 7 years via a facility-based delivery strategy

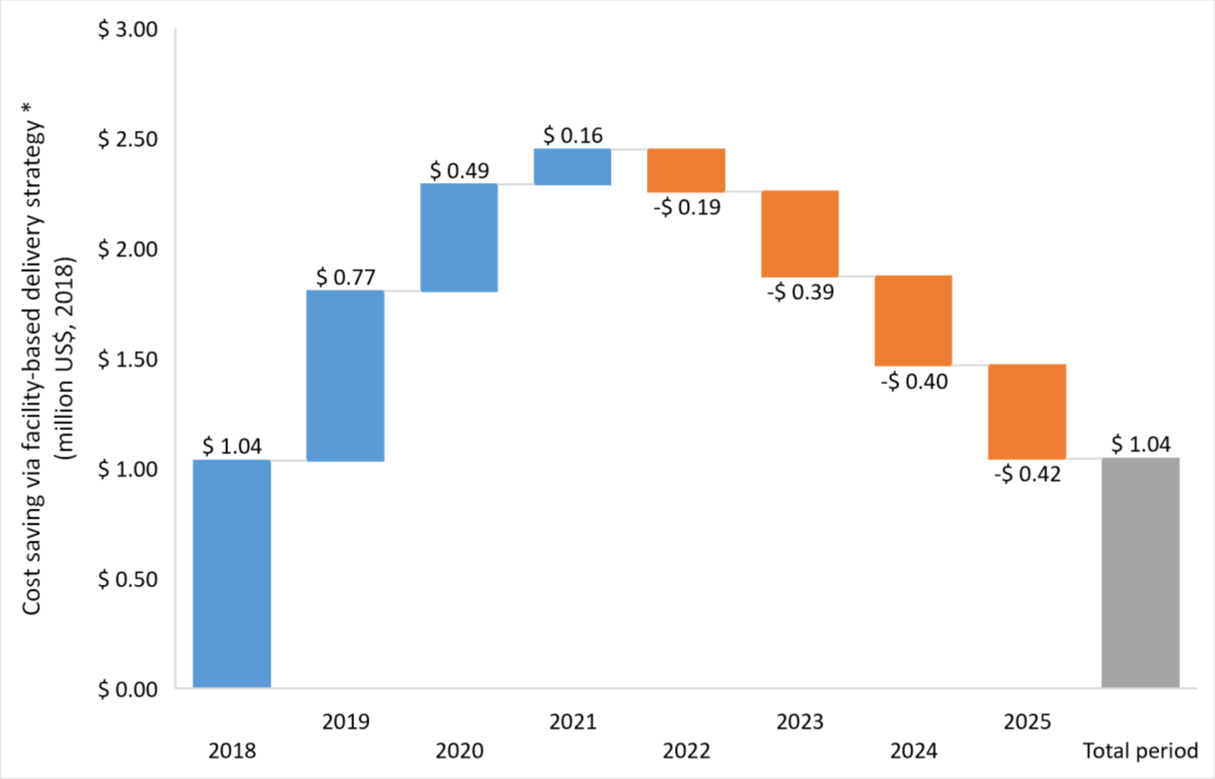

*: Costs were adjusted to 2018 prices using Vietnam's consumer price index. Blue bars represent cost savings (positive amounts of money). Orange bars represent incremental costs when replacing the present schedule of TT to 15-16-year-old girls in high-risk areas and Td response campaigns for children aged 7 years. The grey bar represents the total cost savings over the period 2018–2025.

Supplement to: Mai VQ, Boonstoppel L, Vaughan K, et al. Cost of delivering tetanus toxoid and tetanus-diphtheria vaccination in Vietnam and the budget impact of proposed changes to the schedule. *Glob Health Sci Pract*. 2022;10(2): e2100482.
https://doi.org/10.9745/GHSP-D-21-00482

Supplement Figure 1b. Estimated cost savings from the new schedule for Td vaccine to children aged 7 years via a mixed delivery strategy

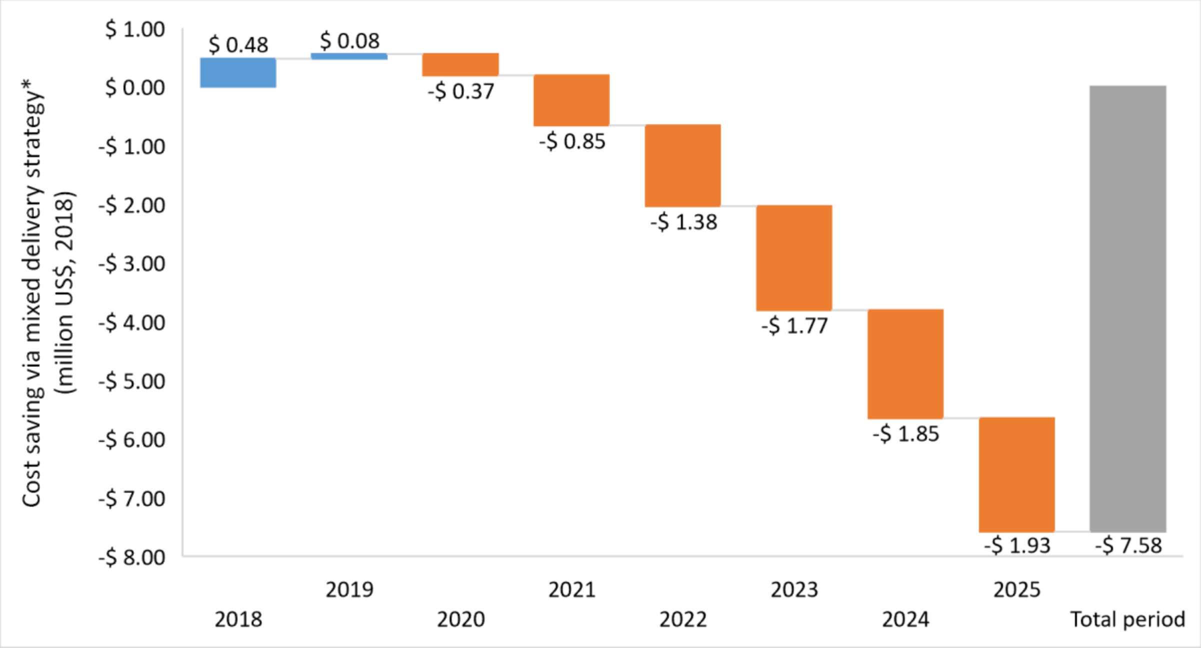

*: The mixed delivery strategy refers to 69% facility-based and 31% outreach vaccination. Costs were adjusted to 2018 prices using Vietnam's consumer price index. Blue bars represent cost savings (positive amounts of money). Orange bars represent incremental costs when replacing the present schedule of TT for girls aged 15–16 years in high-risk areas and Td response campaigns for children aged 7 years. The gray bar represents the total cost savings over the period 2018–2025; because it is a negative amount, the mixed delivery strategy would result in an additional cost as compared to the current strategy.

Supplement to: Mai VQ, Boonstoppel L, Vaughan K, et al. Cost of delivering tetanus toxoid and tetanus-diphtheria vaccination in Vietnam and the budget impact of proposed changes to the schedule. *Glob Health Sci Pract*. 2022;10(2): e2100482.
https://doi.org/10.9745/GHSP-D-21-00482

Supplement Figure 1c. Estimated cost savings from the new schedule for Td vaccine to 7-year-old children via school-based delivery strategy (2018 US$)

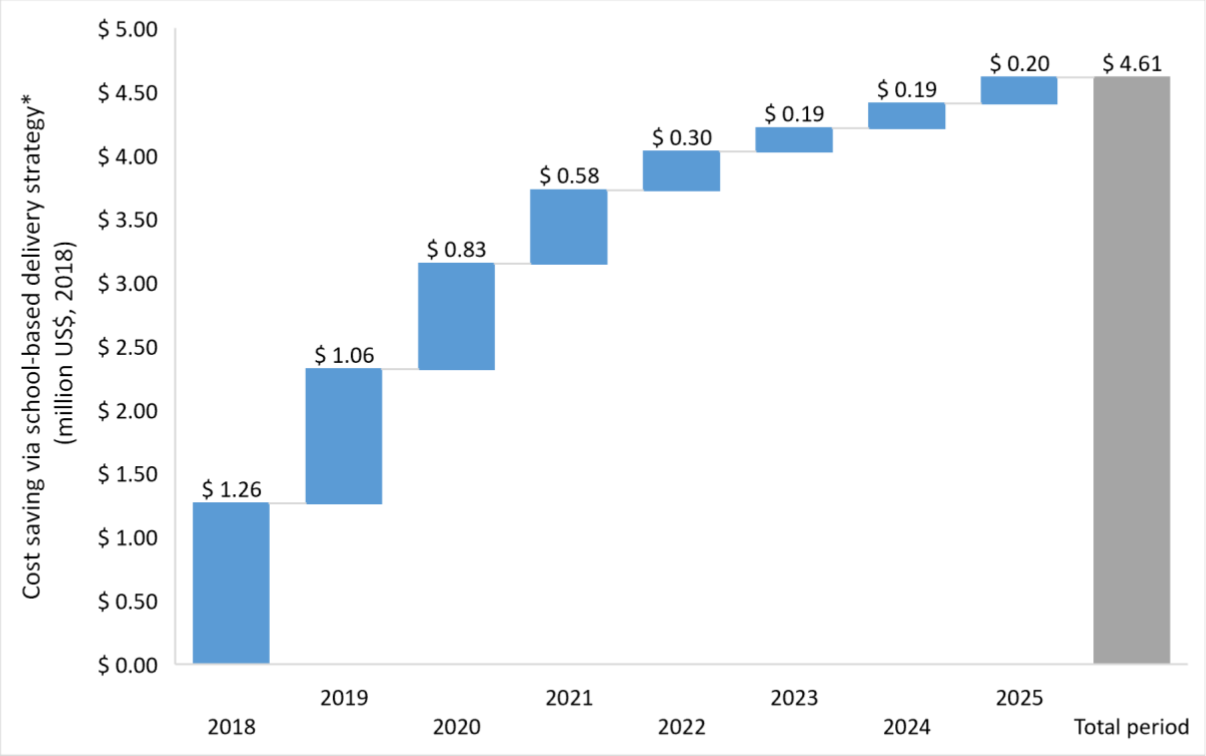

*: Costs were adjusted to 2018 prices using Vietnam's consumer price index. Blue bars represent cost savings (positive amounts of money). The grey bar represents the total cost savings over the period 2018–2025.

Supplement to: Mai VQ, Boonstoppel L, Vaughan K, et al. Cost of delivering tetanus toxoid and tetanus-diphtheria vaccination in Vietnam and the budget impact of proposed changes to the schedule. *Glob Health Sci Pract*. 2022;10(2): e2100482.
https://doi.org/10.9745/GHSP-D-21-00482

Supplement Figure 2a. Budget impact of Td replacement during 2018-2025 with a 7% increase in health worker basic salaries

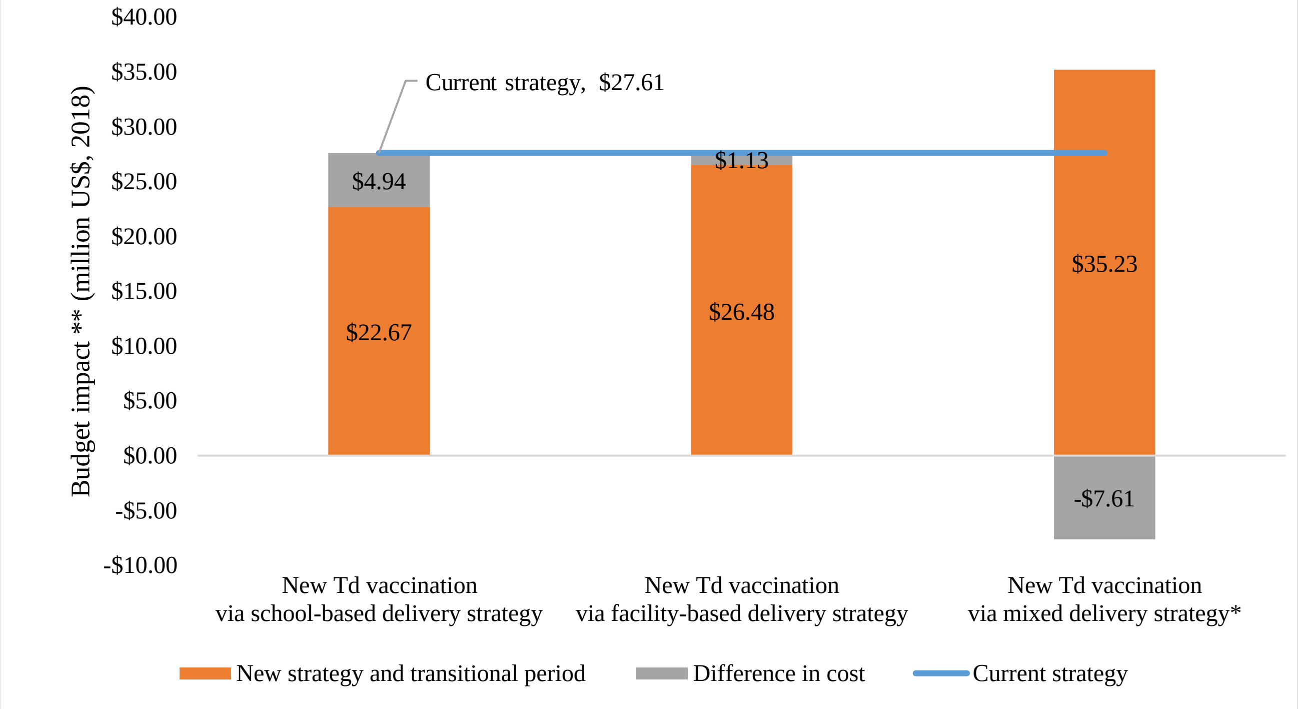

*: The mixed delivery strategy refers to 69% facility-based and 31% outreach vaccination.

**: The costs/dose in 2018 adjusted by 7% increase in staff basic salaries were US$1.97, US$4.09, US$ 1.68, US$3.66 for facility-based, outreach, school-based, and campaign delivery, respectively. Costs/dose in 2019-2025 were calculated to increase 4% annually according to Vietnamese inflation rate. Estimated number of Td doses to be delivered in the new plan was 1,111,000 in 2018. Number of doses for years 2019–2025 increased by 1% according to Vietnamese population growth rate.

Supplement to: Mai VQ, Boonstoppel L, Vaughan K, et al. Cost of delivering tetanus toxoid and tetanus-diphtheria vaccination in Vietnam and the budget impact of proposed changes to the schedule. *Glob Health Sci Pract*. 2022;10(2): e2100482. https://doi.org/10.9745/GHSP-D-21-00482

Supplement Figure 2b. Budget impact according to the Td replacement during 2018-2025 with full coverage for staff' allowance

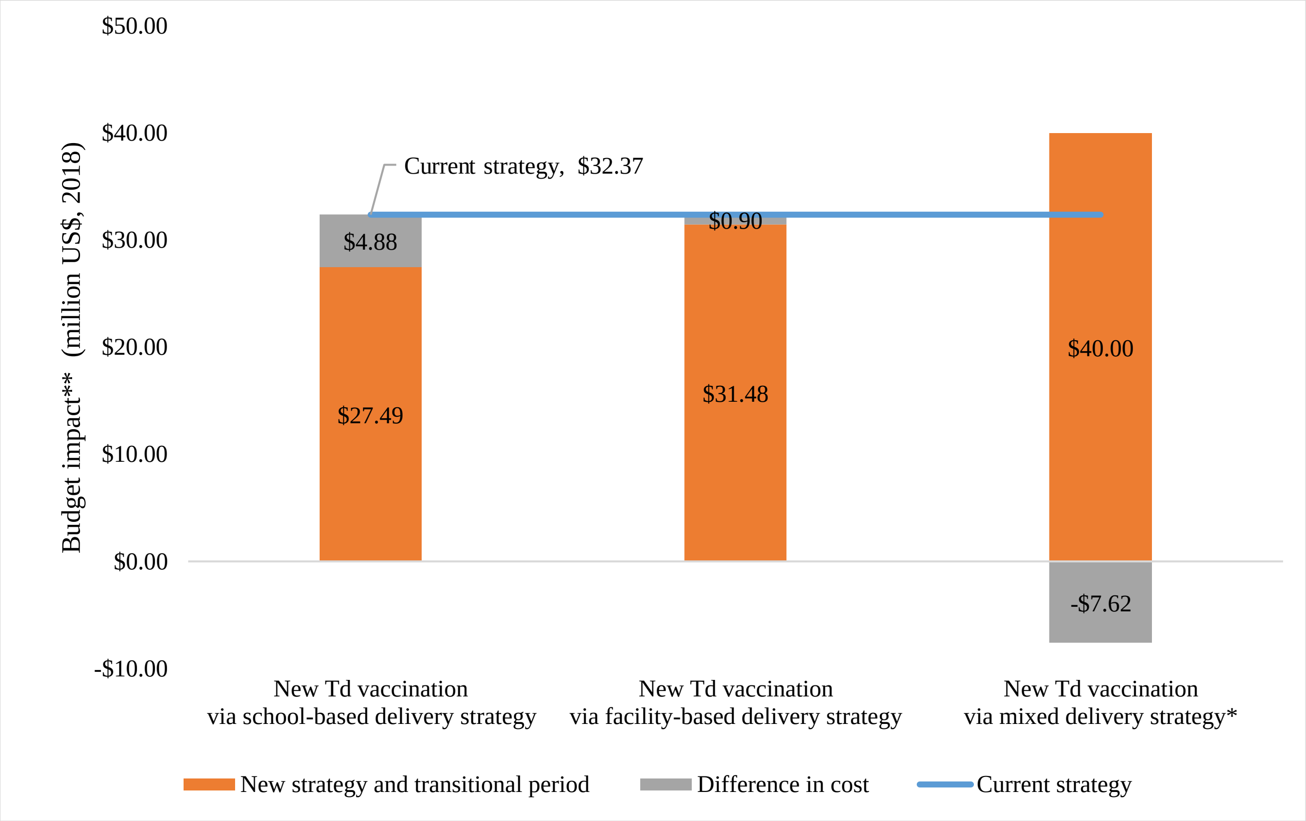

*: The mixed delivery strategy refers to 69% facility-based and 31% outreach vaccination.

**: The costs/dose in 2018 adjusted to cover all staff allowances were US$2.34, US$4.41, US$2.04, US$4.23 for facility-based, outreach, school-based and campaign delivery, respectively. Costs/dose in 2019-2025 were calculated to increase 4% annually according to Vietnamese inflation rate. Estimated number of Td doses to be delivered in the new plan was 1,111,000 in 2018. Number of doses for years 2019–2025 increased by 1% according to Vietnamese population growth rate.

Supplement to: Mai VQ, Boonstoppel L, Vaughan K, et al. Cost of delivering tetanus toxoid and tetanus-diphtheria vaccination in Vietnam and the budget impact of proposed changes to the schedule. *Glob Health Sci Pract*. 2022;10(2): e2100482.
https://doi.org/10.9745/GHSP-D-21-00482

Supplement Figure 2c. Budget impact according to the Td replacement during 2018-2025 with an annually 10% reduction in Td transitional period

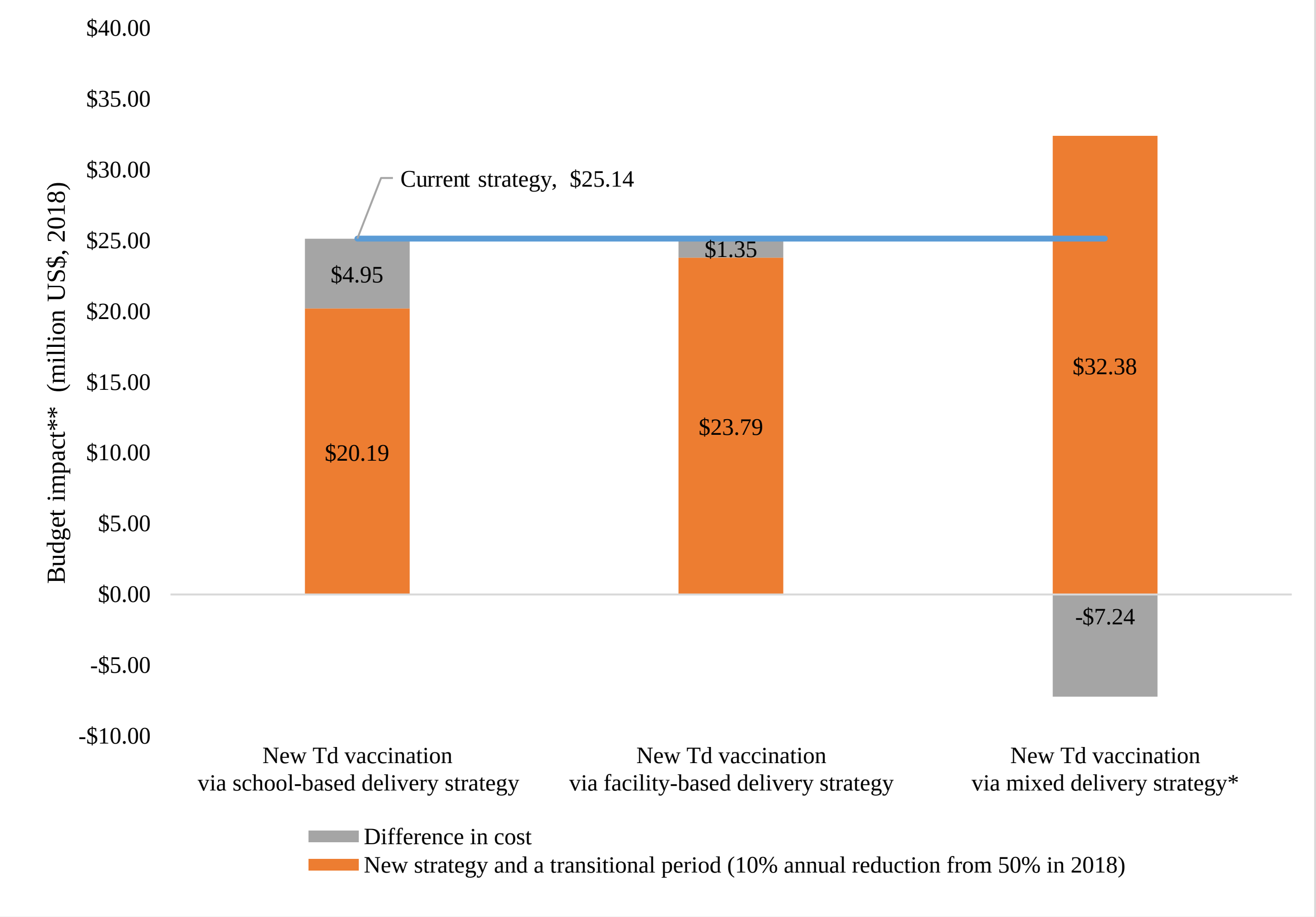

*: The mixed delivery strategy refers to 69% facility-based and 31% outreach vaccination.

**: Costs/dose in 2019-2025 were calculated to increase 4% annually according to Vietnamese inflation rate. Estimated number of Td doses to be delivered in the new plan was 1,111,000 in 2018. Number of doses for years 2019–2025 increased by 1% according to Vietnamese population growth rate. The transitional period for diphtheria response campaign was reduced 10% annually from 50% in 2018 to 0% in 2023.
